# ICT, Disability, and Motivation: Validation of a Measurement Scale and Consequence Model for Inclusive Digital Knowledge

**DOI:** 10.3390/ijerph18136770

**Published:** 2021-06-24

**Authors:** Marta Medina-García, Lina Higueras-Rodríguez, Mª del Mar García-Vita, Luis Doña-Toledo

**Affiliations:** 1 Deparment of Pedagogy, University of Jaén, 23071 Jaén, Spain; mameding@ujaen.es; 2 Deparment of Education, University of Almería, 04120 Almería, Spain; margvita@ual.es; 3 Marketing and Market Research Department, University of Granada, 18071 Granada, Spain; luisdt@ugr.es

**Keywords:** ICT, disability inclusion, digital competence, inclusive digital knowledge, PLS

## Abstract

The use of ICT (information communication technology) as an educational resource is becoming more evident in the education systems of most countries, even more so with the COVID-19 crisis. When it comes to disability and education, ICT becomes a tool for social and educational inclusion. This study presents the validation and evaluation of a measurement scale on ICT literacy for inclusive education. In addition, based on previous literature, a conceptual model is proposed and validated through PLS (partial least squares) using a sample of 142 teachers from all educational stages. The results show that teachers’ ICT knowledge to ensure inclusion consists of five dimensions on specific needs. ICT knowledge has a positive impact on teacher motivation and ICT use. Teachers at primary and early childhood education levels have a lower motivation and use of ICT, although they have a higher knowledge of disability. The results found allow progress to be made in measuring the educational inclusion of schools and the ICT knowledge needed to ensure care and support for all people. A notable implication is the need for training on ICT and disability within educational policies.

## 1. Introduction

Information communication technology (from now on ICT) is a fundamental element of modern society [1,2]. The use of ICT in educational processes has grown considerably in recent times [3,4], especially in the wake of the COVID-19 pandemic, making it possible to reach larger sections of the population, including disadvantaged groups, and thus contributing to democratic education [5].

The use of ICT becomes a fundamental axis for the achievement and fulfilment of the sustainable development goals. According to Huawei research on the relationship of ICTs in the development of SDGs (sustainable development goals) [6], goals with the highest correlation with ICTs include SDG 9: infrastructure, industrialization, and innovation; SDG 3: good health and wellbeing; and SDG 4: quality education. Specifically, for the area that concerns us, the implementation of ICT is key to guaranteeing quality and inclusive education, as stated in SDG 4.

The importance and awareness of ICT often lies at the heart of education, enhancing the teaching and learning process [1,5] and providing new opportunities for learning [7]. ICT can be a useful resource to make content more interactive and engaging for learners and to improve the quality of materials [3,5]. Hence, it is important for teachers to incorporate technology in their teaching to generate benefits for their students [1]. 

Inclusive education involves changing traditional models of teaching [8]. Didactics is relevant in education at all stages, primarily to promote success for traditionally excluded students [9], such as students with disabilities. Research such as that of [10] indicates improvements in accessibility for students with disabilities by combining special methods and materials with the use of ICT.

Depending on the type of disability that students present, they encounter different barriers, and these are eliminated by providing alternative forms of learning to students who can learn in different ways and that the teacher must be aware of them [11]. There are various technical aids to incorporate technologies, and each of them is aimed at the accessibility of a certain type of disability (visual, auditory, motor, cognitive) [12].

The COVID-19 pandemic and home confinement have brought about many changes worldwide [13], including at the social and educational level, transforming social relations and teaching and learning processes, taking on the challenge of implementing new methodologies to guarantee learning and its quality while ensuring health safety [14,15,16,17]. The rise of distance learning is significant, with the student working online from home and the teacher conducting work both digitally and remotely [18] being the most effective method to ensure continuity of education during this socio-health crisis [19]. Against this backdrop, the use of ICT in an innovative way has gained momentum worldwide to meet the educational demands of the current pandemic [20,21]. The distance learning process and the widespread use of ICT have become a preference for educational institutions [19] and a challenge for teachers and students to achieve educational goals and the success of the whole student body [22].

When it comes to disability and education, ICT becomes a tool for social inclusion. Previous studies have shown that educational institutions, together with their community, should provide teachers with high-quality disability training that is supportive in terms of social and material resources, which significantly enhances positive attitudes towards intellectual disability for the improvement of inclusion processes for this population [23]. Therefore, increased opportunities for teacher involvement in the social environments of people with disabilities broaden learning possibilities by ensuring mechanisms for people with disabilities to act as causal agents in their communities [24]. Within these tools, ICT promotes social inclusion by enabling access to information, knowledge, and the learning process, and are essential to ensure accessibility [25]. It is important that digital resources show their accessibility functions to promote inclusive educational practices and for teachers to be aware of the difficulties and challenges of ICT accessibility [26], since the results of some research show the low skills and low level of training and knowledge presented by teachers in the use of ICT with students with disabilities [27,28].

As indicated above, to use ICT in an inclusive classroom context, attitudes and teacher training around ICT are essential to foster learning and promote educational innovation so that teachers are technologically qualified, pedagogically trained, and, above all, empowered [28]. Deficits in teacher training in ICT and accessibility can be addressed by including them in continuous teacher training programs [25], since, as Medina-García, García-Vita, and Higueras-Rodríguez [29] point out, it is necessary to promote training actions aimed at knowledge in the field of disability, considering the shortcomings present in teachers. Likewise, this issue could be tackled by including these topics in the curricula themselves [26].

Education, ICT, and the inclusion of persons with disabilities thus become priority issues. Incorporating ICT into the education system means spreading the “culture of accessibility” and moving towards ensuring the educational inclusion of students with disabilities [26]. All of this is within the framework of the fundamental precepts offered by the right to inclusive education, which include offering quality education for all students [30,31]. This implies the right of all learners, including learners with disabilities, to access and use the same learning materials and ICT [32]. While ICTs are important for the development of our lives in general, for people with disabilities, ICTs mean having autonomy in their lives and in their educational processes. The teaching and learning processes mediated by ICT at any educational stage must be carried out with guarantees in terms of accessibility, which means highlighting the training of teachers in a way that brings together two fundamental aspects: ICT and disability.

Under these premises, we propose this study with the following objectives:

(1) To validate and evaluate a scale about ICT knowledge applied to inclusive educational needs

(2) To assess the degree of inclusion among teachers 

(3) To validate a background model of the use of technologies in the classroom.

## 2. Proposal of a Conceptual Model on Knowledge, Motivation, and the Use of Technology: Literature Review

The proposed model is structured with five hypotheses based on the review of the elements that make up ICT in disability issues, as well as the relationship between teacher training in disability, their motivation, the use of technologies, and the different educational stages.

ICT enables people with disabilities to be included in their environment by removing barriers of various kinds [25]. This affects the educational field, as students with disabilities face different accessibility difficulties that can be solved thanks to the use of ICT in the classroom [33], for which it is necessary for teachers to have knowledge on the subject [27], as it conditions the use and attitude towards them. In the case of the application of ICT to people with disabilities, the lack of knowledge is an aspect pointed out in the scarce research on the subject, even though ICTs are emerging as fundamental elements to guarantee the inclusion of people with disabilities [34].

Likewise, research on the subject shows, on the one hand, the potential of ICT in the education of students with disabilities [35], the importance of teacher training in ICT to ensure educational inclusion [11], and a scarce knowledge of teachers on the use of ICT for people with disabilities [27].

These aspects related to knowledge and ICT allow us to establish the first hypothesis of this research:

**Hypothesis** **H1.** *Knowledge about ICT and disability is a second-order construct composed of specific knowledge about motor, hearing, visual, cognitive, and accessibility needs.*

Another key aspect of this work relates to the use and motivation of ICT by teachers. To implement the use of ICT in schools, the work of the teacher is important. Specifically, motivated teachers show greater use of ICT in their classes [36]. The elements that motivate the use of ICT in education are mainly training, lower level of effort for implementation or attitudes [37], and motivation for computer science and the use of ICT support [38]. However, knowledge alone is not sufficient to promote teachers’ motivation to use ICT in their classrooms [39,40,41]. 

Overall, research addressing teachers’ motivation to use ICT is scarce. However, findings on the subject indicate that motivation is a relevant factor in implementing their use in teaching and learning processes. Specifically, the elements of motivation reported by teachers to use ICT are the following: the perceived ability to use ICT; difficulties experienced in using ICT; the level of resources available and their satisfaction with ICT; and whether the use of information technology in teaching is considered interesting and enjoyable. Ultimately, teachers consider that making lessons more enjoyable contributes to the improvement of learning processes [42].

In view of these issues, it is necessary, on the one hand, to implement changes that increase teacher motivation to improve the level and quality of ICT use [36]. On the other hand, it is necessary to show evidence for the motivational factors that influence the use of ICT [41,42].

Based on the literature reviewed, the following hypotheses are proposed for this research:

**Hypothesis** **H2.** *Knowledge of ICT applied to people with disabilities has a positive impact on teacher motivation.*

**Hypothesis** **H3.** *Knowledge of ICT applied to people with disabilities has a positive impact on the use of technology in the classroom.*

**Hypothesis** **H4.** *The use of technology increases teacher motivation.*

Finally, we need to address the differences that exist around ICT and its use at different educational stages. Teachers and lecturers in the middle stages use ICT with the aim of making the learning process more attractive, more effective, and easier. Therefore, training and improving teaching competences involves knowledge about ICT [43].

Wikan and Molster’s [44] research on Norwegian secondary school teachers and ICT indicates that, although the majority show commitment to its use, they do not see its educational value, except in increased motivation and greater access to material. This issue translates into a lack of confidence in ICT. Furthermore, despite the significant benefits for the educational community, secondary school teachers in the study point to difficulties in accessing the internet, lack of technical support, and lack of time in class as barriers to implementation [45].

Finally, a noteworthy aspect of this educational stage is that trainee teachers are poorly trained and prepared to use ICT in a didactic way, despite having technical skills [46].

In the case of higher education, the use of ICT is essential [46]. Specifically, in Southeast Asia, the integration of ICT at this stage has been a key element in addressing the various challenges of the education system [47,48]. The use of ICT in the learning process is viewed favorably by university students [49], and even increases their motivation level [50].

In short, teachers at all educational stages must acquire digital competences to use ICT in an optimal way, considering the abilities of their students to develop their didactic work appropriately [51]. Likewise, at all stages, the implementation of ICT should be done considering the educational needs of the students and the educational context as part of a relationship of general strategies [47]. Based on all of the reviewed research, the last hypothesis of our study is put forward:

**Hypothesis** **H5.** *The stage of teaching has a moderating effect on ICT knowledge and its effect on motivation and use of technology.*

## 3. Proposed Model

This model is designed to provide a concise and precise description of the experimental results, their interpretation, and the experimental conclusions to be drawn.

**Figure 1:**
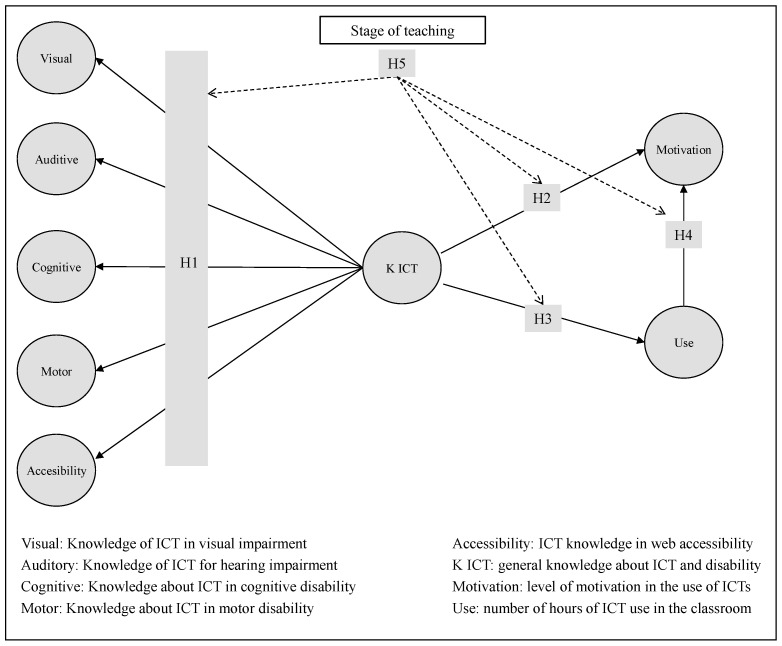
Proposed conceptual model.

Figure 1, following the literature review and the hypotheses put forward, presents the conceptual model to be tested. The model considers that general ICT knowledge is formed and reflects knowledge about specific needs: hearing, motor, visual, cognitive, and web accessibility disabilities. General knowledge has a positive impact on motivation and technology use, and this again has a positive impact on motivation.

It is important to note that hypothesis 1 is formulated as a confirmatory factor analysis of the dimensions that make up general knowledge about ICT and disability. This is a formative relationship of general knowledge based on knowledge of specific aspects, such as visual or hearing needs, all of which are complementary to each other. Specific knowledge about certain disabilities represents manifestations of such knowledge, i.e., having general knowledge about ICT and disability.

## 4. Method

### 4.1. Sample

Data collection was carried out through a self-administered online survey. It was carried out during the months of February and March 2020. The target population was all teachers in the educational field, from the preschool to university level of Spain. The sample collection was completed on 24 March, in the middle of strict confinement due to COVID-19 in Spain. The final sample reached 142 individuals after the relevant data cleaning. Individuals were selected by non-probability snowball sampling from the questionnaire dissemination. Once completed, they were asked to provide contact details of other interested parties or to forward the survey to others. There was no sampling frame or sampling list, given the size of the population to be analyzed from the entire teaching population. Therefore, the teachers who participated freely and the refusal rate of the questionnaire are not available. The error under the assumptions of infinite sample simple random sampling (*p* = q = 0.5; z2 = 1.96) was 5.9%. Therefore, the margin of error is small, and the representativeness of the sample is confirmed.

To reduce the dropout rate, information was inserted about the purpose of the research, the university and the researchers were identified, it was stated that there were no right or wrong answers, only the opinion and experience of the respondents, and the anonymity of the individuals was assured, as well as the protection of data and the non-use of the data for other purposes. 

Responses that took less than 4 min to complete (average time was 8 min) were eliminated. Likewise, answers in which patterns of response were found through the Mahalanobis method of calculating distances and with the same response in all questions were eliminated.

Finally, 72.5% of the sample was made up of women and 52.1% had less than 10 years of teaching experience; 33.1% used more than 50% of their time in the classroom for technology; and 35.9% taught at the primary level, compared to 12% in vocational training and 19.7% at the university level. Younger teachers accounted for 37.5% of the sample (under 36 years of age), while those over 45 years of age represented 29.6% of the sample. Most of the sample came from the southern Spanish region of Andalusia (59.5%). Table 1 shows the characteristics of the sample.

### 4.2. Questionnaire and Measurement 

An online questionnaire consisting of three main parts was administered through a Google Forms application: (1) use, motivations, and opinion about ICT and disability; (2) knowledge about ICT and different disabilities; (3) socio-demographic characteristics of the respondents (age, gender, stage at which they teach, years of experience, etc.).

To measure knowledge of ICT and disability, an adapted version of the scale proposed by Cabero, Fernández, and Córdoba [34] was applied. This instrument by Cabero et al. [34] is the only attempt to develop a specific diagnostic instrument on ICT and disability. This work presents an important novelty and advancement with respect to such an instrument: it is applied to teachers, instead of teachers in their initial training, and its validity and reliability were tested. In addition, a shortened version is proposed. The items were selected using the Delphi method, whereby, in two waves, they were assessed by three university professors of education. After the first rating, each expert had access to the average score of each item (on a scale from 0 = not at all important to 10 = very important) before proceeding to the second voting. The items with the highest mean importance score and the lowest standard deviation were selected. Finally, the questionnaire consisted of 30 items (see Appendix A) divided into the six dimensions of the original scale by Cabero et al. [34], composed of 65 items. All statements were rated according to a five-point Likert scale, with 1 = strongly disagree and 5 = strongly agree.

Regarding the questions referring to motivation when choosing ICT, these were measured through a Likert metric question, for which the respondent indicated from 0 (not at all) to very much (10). Finally, regarding the percentage of time spent using ICT in the classroom, this was a ratio metric scale where the percentage spent by the respondent was indicated.

### 4.3. Data Analysis

To analyze the data obtained from the survey and assess the relationship between the constructs, structural equation modelling with PLS (partial least squares) using Smart PLS 3 software was used to test the validity and reliability of the models. PLS has begun to be used as a technique in the field of education, as demonstrated in the study by Ghasemy et al. [52]. 

Compared to other covariance-based tools (CBM), such as Lisrel or Amos, PLS is a powerful analysis method that has recently been developed as an alternative to these methods [53,54]. The objective of PLS is to estimate dependent variables, maximizing their explained variance. In our case, bootstrapping was performed with 5000 subsamples for both the global model estimation and for each of the different subsamples of the multigroup analysis. The multigroup analysis dealt with the comparison between those who taught as teachers (primary and early childhood) and the rest of teachers at higher levels (secondary, vocational training, and university).

The confirmatory factor analysis of the reduced scale proposed by Cabero et al. [34] was carried out through the procedure admitted by PLS: checking the validity and reliability of the constructs through a second-order confirmatory model, formed by all of the items that make up the dimensional scale proposed to measure the specific aspects of ICT and disability.

## 5. Results 

Before testing the proposed model, the means of the factors for which respondents claimed to have greater knowledge were reviewed. Respondents had the least knowledge about web accessibility (M = 1.91), while the most knowledge was about hearing impairment (M = 2.21). Thus, no knowledge about specific disability exceeded the midpoint of the scale from 1 to 5. On the other hand, motivation among the sample was high (M = 7.78 out of 10), and the use of technology in class on average was 40%.

Secondly, concerning the measurement model of the proposed model, Table 2 shows the psychometric properties of the scales. Four items were eliminated, as they did not present adequate psychometric properties, which can be found in Annex 1 (VISUAL1, AUDITIVE2, AUDITIVE5, and MOTOR5). All loadings were significant both in the overall model and in the two subgroups according to teaching stage (*p* < 0.01), and were higher than 0.7 [55]. Cronbach’s alpha, composite reliability (CR), and average variance extracted (AVE) values were above acceptable cutoff levels (0.7, 0.8, and 0.5, respectively) [56,57]. It can therefore be concluded that the scales used have good psychometric properties in all cases.

On the other hand, discriminant validity was tested by applying the procedure proposed by Fornell and Lacker [53], whereby the square root of the variances extracted must be greater than the correlations between the constructs. Table 3 shows the results achieved for the overall model. It is important to note that, being a confirmatory factor model with respect to general knowledge, it was corroborated that the correlation was equal or higher for the relationship between the second-order construct and each of the specific disability dimensions. In the different groups according to teaching stage, the values were practically similar and valid in all cases.

Finally, the model fit provided by the PLS standardized root mean square residual (SRMR) was 0.02. A value below or equal to 0.08 is adequate for PLS path models in strict criteria [58] and below 0.10 in more flexible criteria [59]. 

The results show (see Table 4) that, referring to the total sample of teachers from the different educational stages, knowledge about disability and ICT was composed of five specific dimensions about disability: motor, cognitive, hearing, visual, and web accessibility (*p* < 0.01 in all relationships). Therefore, increasing knowledge about any specific aspect reciprocally enhances teachers’ capacity and ability by increasing their knowledge.

Secondly, knowledge about ICT and disability had a positive impact and effect on both the increased use of technology as a teaching resource and on the teacher’s own motivation. Therefore, both hypothesis 2 (β = 0.167; *p* = 0.01) and hypothesis 3 (β = 0.163; *p* = 0.04) were confirmed. Finally, according to what was hypothesized in H4 (β = 0.574, *p* = 0.00), the increased use of technology as a resource had a positive effect on teachers’ motivation to be trained on the technological needs of people with disabilities and general accessibility. A positive virtuous circle occurred, where improved ICT skills increased teacher satisfaction and motivation.

Next, the multi-group analysis (which also supports the measurement model) was carried out in order to discern whether there were differences according to the stage in which the teacher worked, i.e., whether he/she was a teacher or a teacher of higher levels (see Table 5).

The results obtained show that there were no significant differences in the relationships of the conceptual model between teachers at the different stages (significance level at 10%). 

However, a relevant aspect of the results was that the relationships between ICT knowledge and disability did not have a significant effect on either motivation (Hypothesis 2 of the conceptual model proposed) or the use of this resource in the classroom (Hypothesis 3). Therefore, teachers were less motivated and used it less, although they had more knowledge about disability in general. Hypothesis 5 on the mediation of the teacher’s educational stage was therefore partially confirmed, as it was not significant in all relationships. Three of the hypotheses raised and discussed in this section were confirmed. The main result, therefore, was that the model was not confirmed among the population of early childhood teachers.

## 6. Discussion

The implementation of ICT in the classroom has meant a before and after in educational processes. This is reflected, even more so, in the health situation of COVID-19, as the use of ICT has been fundamental to carry out teaching at different educational levels. Despite its benefits, the evolution of ICT development in education has been slow and laborious [60]. As Ali [56] mentions in his research, the level of satisfaction with the use of ICT is high. Their implementation encourages active learning and aids problem solving. All of this opens possible avenues for further research [16].

The results obtained in this research allow inferences to be drawn about the use of ICT in the classroom at different educational stages, i.e., its implementation is enriched from the basic stages, such as early childhood and primary education, to the university stage. This enrichment has been demonstrated in this work in the positive effect of ICT on motivation, as well as the importance of knowledge in adequately attending to students with disabilities.

In our results, the stage where these tools are most used is in higher educational stages. Research by Fernández-Batanero, Graván, and Rojas [28], Konstantinos, Andreas, and Karakiza [61,62], and Ramírez-Rueda et al. [63] corroborate the results obtained and add that their use is reinforced by teacher training in ICT, their motivation, and their predisposition to learn. Likewise, our study indicates that the infant and primary education stages are stages where the use of ICT is more limited, either due to the interpersonal skills of the teachers themselves [64,65] or due to their interest and motivation towards ICT [66,67].

In the case of university higher education, the results of Nae’s research [60] point to aspects such as deficits in technological knowledge, lack of institutional support, lack of awareness of the educational outcomes promoted using ICT, and the very traditional nature and pedagogy of the university institution itself, which may explain the disinterest and apathy of its implementation in the Japanese higher education system. However, some research, such as that of Ali [61], points to a change in perspective, and reveals that more universities are implementing online learning and, therefore, the use of ICT. In this sense, they point out that the factors that affect ICT learning are resources, training, security, accessibility, and motivation. In short, we can say that, for ICT to be implemented in higher education in an optimal way, the training and motivation of teachers is fundamental, something that corroborates the results found in this work. This is reaffirmed by Yuen and Ma [68] when they pointed out that, to implement ICT in the learning process, teachers need to gain confidence in ICT.

If we focus on the early childhood stage, the controversy about the appropriateness of children’s exposure to ICT is still present. The results of Kim’s research [69] show that the implementation of online learning has meant that early childhood teachers have had to reflect on how to optimize the teaching and learning process with these tools. The demands of our century mean that children must acquire ICT competences, therefore, teachers must implement these issues in their teaching development [70,71,72].

One of the reasons that may explain the deficits of early childhood teachers in the use of ICT may be a gap in their training in relation to the level of digital competence that currently prevails [73]. Another reason that strengthens and justifies these results is the importance of manipulative aspects at this stage, as well as the rise of alternative pedagogies characterized using natural and simple elements present in the child’s immediate environment, as is the case of the Montessori method or didactic strategies, such as manipulative mathematics, among others. These educational trends mean that the use of ICT in the infant stage is scarce in comparison with other educational stages. This issue coincides with Kim [64] when he points out that the development of ICT in early childhood implies the implementation of methodological strategies that are not very appropriate for the demands and needs of children of this age. This may explain the result found in our model that a greater knowledge of ICT does not increase motivation and use among teachers. However, there are ICT resources that can be useful for this educational stage if teachers use them appropriately.

To combat this issue, Mama and Hennessy [74] and Somekh [75] propose the development of novel and attractive training actions that increase motivation and use of ICT. Such training actions for early childhood teachers have not addressed how to develop the online teaching process, but the reality of COVID-19 makes this issue a training priority, so that training programs need to address ICT competence in more depth [69].

Another fact to be highlighted in our research is that teachers in the infant and primary education stages have more knowledge about disability. We understand that this is because they have more solid training in pedagogy and didactics in relation to educational attention to the diversity present, in this case, in people with disabilities. This lack of training in our findings is based on the OMS (World Health Organization) [76], which considers the need to improve and train teachers at all educational stages to address the curricular barriers that exist for people with disabilities. In this sense, to combat the results of our research, Guasch and Hernández [77] point out the need to provide our teachers with more innovative training and perspective based on the principles of universal design for all people, which implies, among other things, changes in didactics to make the teaching and learning processes accessible.

Another interesting fact is that most of the teachers who use ICT in their classrooms have less than 10 years of professional experience. Based on the research of Escudero, Martínez-Domínguez, and Nieto [78], continuous teacher training is a key factor for the integration of ICT in the classroom. Professional development programs need to be connected to the socio-educational reality, and there needs to be a connection between theory and practice. To these factors, we can add those pointed out by Vrasidas [79], who points to difficulties in the availability to organize classes and an inadequate curriculum as a barrier to the implementation of ICT despite the school having adequate ICT resources.

On the other hand, our study shows that there is less knowledge about web accessibility, but more knowledge about hearing impairment. These results are like those found in other previous studies, such as those of Fernández-Batanero et al. [80].

The results obtained allow us to infer that the introduction of ICT resources, as well as the teaching methods and strategies associated with them, do not displace traditional resources, but lead to hybrid or mixed models, in which both types of resources coexist. In this sense, teachers’ engagement with ICT is related to recognizing the importance of education and relying on the virtues of technology [16].

There is no doubt that the use of ICT in educational processes is appropriate and relevant; it makes learning more agile and easier, and, above all, functions as a complement to constructivist learning, especially when it comes to students with learning difficulties. However, it is important to bear in mind their limits when they generate a negative result or when they do not contribute to eliminating the different types of barriers faced by these students [10]. In this regard, we must keep in mind that it takes commitment and sacrifice to make ICT accessible to persons with disabilities. According to Hattangdi and Ghosh [5], the success of ICT development depends on many issues, including training, but training should be directed towards pedagogical rather than technical issues.

In short, if we want to achieve sustainable education, we must consider all people, including people with disabilities, who have traditionally been excluded from education. For this reason, teachers must be aware of the use of ICT and take advantage of it as an appropriate resource for the educational and social inclusion of people with disabilities.

## 7. Conclusions

This work presents an important novelty in using ICT for the educational inclusion of people with disabilities. The originality of this topic advances as this research develops, observing the deficits present in the literature on the approach to this topic, and how we can continue to advance in this direction. The study also represents an important advance at the methodological level, as it offers for the first time the complete validation of a scale of ICT knowledge at all educational stages and confirms a background model of ICT use and motivation among the teaching staff. An important new development is the validation of an ICT knowledge scale. In addition, the model presents as main findings:

- ICT and disability knowledge are shaped by five dimensions: knowledge about motor, hearing, cognitive, visual, and accessibility needs
- Knowledge about ICT increases teacher motivation
- Knowledge about ICT has a positive effect on the use of technologies in the classroom
- The use of ICT increases teacher motivation

ICT is a medium for learning. Both teachers and students, as well as any professional in the field of education, use it as a medium for the teaching and learning process. As it is such an effective tool, it can be used at different educational stages. In short, we estimate cause and effect relationships, i.e., that ICT knowledge is a cause and has a positive effect on teacher motivation and the use of ICT and the mediating character of the educational stage.

In conclusion, the “big ideas” that resulted from this research are related to: (a) identifying the educational stages where the use of ICT as an educational tool for the inclusion of people with disabilities is present; (b) knowing the barriers and limitations that teachers of the different educational stages have for the use of ICT in their classrooms; (c) knowing the level of motivation of teachers in reference to ICT; and (d) presenting the deficits in knowledge and training present in teachers in terms of educational attention to students with disabilities.

Along these lines, we can affirm that the use of ICT in the classroom improves the teaching and learning process, in turn promoting the inclusion of all pupils. All of this could be improved with greater teacher training in relation to ICT knowledge and its applicability at the different educational stages. However, this would be possible by improving training plans, both at the initial level (university) and at the continuous level (in-service training).

## 8. Limitations of This Study

This is a causal study in the Spanish educational context. In our case, we have carried out structural equation modelling through PLS (partial least squares). Structural equation modelling (SEM) is a multivariate statistical technique for testing and estimating causal relationships from statistical data and qualitative assumptions about causality. This research is supported by international research on what teacher training should be like in relation to ICT, and its promotion to improve learning for people with disabilities. There are limitations about the bibliography on our subject: teacher training and motivation of students with disabilities in the use of ICT in the different educational stages require further study. Likewise, these shortcomings are reflected in the scarcity of works that address issues such as accessibility and disability as a barrier to the implementation of ICT and a key element for generating equity in educational processes and guaranteeing educational inclusion.

For this reason, it would be advisable to continue researching this subject at different educational levels, delving deeper into different aspects and competences to find out more specifically how teachers are trained in this regard. Likewise, we consider it interesting to carry out a comparative study at the European level that will lead us to obtain data not only in relation to the differences, but also in terms of cases of good practice that will serve as a horizon for their implementation in our country.

At the methodological level, the measurement of motivation was performed by means of a single item and the invalidation of four measurement scale indicators. On the other hand, no information is available for those who refused to participate in the research.

Finally, about the practical implications derived from the study, it is worth highlighting the need to include a universal design for learning and training in curriculum design for all people in university curricula, as well as in the pedagogical training offered for didactic training aimed at teachers in higher and university stages. The transfer of our findings to the reality of education provides educational managers with evidence to support and reinforce the right to inclusive education for all students through useful educational policies that guarantee this right.

## Figures and Tables

**Table 1:** Sample characteristics.

Variable	Description	Frequency	%
Sex	Men	39	27.5
	Women	103	72.5
Age	25–35	53	37.3
	36–45	47	33.1
	>45	42	29.6
Region	Andalusia	83	58.5
	Rest of Spain	59	41.5
Stage of teaching	Preschool Education	19	13.4
	Primary Education	51	35.9
	Secondary Education	27	19.9
	Professional training	17	12.0
	University	28	19.7
Percentage of technology use in the classroom	On 0 and 25	44	31.0
	26–50	50	35.2
	51–70	24	16.9
	71–100	24	16.9
Years of teaching experience	1–5	55	38.7
	6–10	19	13.4
	10–15	27	19.0
	More than 15	41	28.9

**Table 2:** Reliability and convergent validity of the measures.

Variables	Average	Weight	CA	Rho_A	CR	AVE
ICT-Visual Knowledge						
VISUAL1	2.16	0.852	0.896	0.863	0.927	0.762
VISUAL3	2.25	0.837				
VISUAL4	2.39	0.904				
VISUAL5	2.20	0.898				
**ICT-Auditive knowledge**						
AUDITIVE1	2.25	0.901	0.906	0.906	0.941	0.851
AUDITIVE3	2.34	0.922				
AUDITIVE4	2.30	0.928				
**ICT-Cognitive Knowledge**						
COGNITIVE1	2.43	0.939	0.944	0.949	0.957	0.817
COGNITIVE2	2.46	0.940				
COGNITIVE3	2.04	0.863				
COGNITIVE4	2.25	0.923				
COGNITIVE5	2.21	0.853				
**ICT-Motor knowledge**						
MOTOR1	2.25	0.934	0.960	0.960	0.971	0.892
MOTOR2	2.30	0.954				
MOTOR3	2.08	0.957				
MOTOR4	2.14	0.934				
**Knowledge of web accessibility**						
WEBACCESS1	2.11	0.858	0.928	0.930	0.946	0.777
WEBACCESS2	1.82	0.905				
WEBACCESS3	1.96	0.864				
WEBACCESS4	1.84	0.861				
WEBACCESS5	1.86	0.917				
**Use of technology in class**						
USE1	2.20	1.000	1.000	1.000	1.000	1.000
**Motivation in ICT use**						
MOTIVATION1	7.76	1.000	1.000	1.000	1.000	1.000

**Table 3:** Discriminant validity.

Dimensions	Web Access	Auditive	Cognitive	Motivation	Motor	Use	Visual
Web access	0.881						
Auditive	0.705	0.917					
Cognitive	0.723	0.899	0.904				
Motivation	0.310	0.243	0.209	1.000			
Motor	0.690	0.883	0.872	0.196	0.945		
Use	0.248	0.106	0.117	0.602	0.113	1.000	
Visual	0.677	0.894	0.847	0.247	0.831	0.157	0.873

**Table 4:** Estimated model results.

N°	Hypothesis	Standardized Coefficient	*p*-Value	Hypothesis Result
WEB Accessibility -> ICT Knowledge	H1	0.237	0.000	Confirmed
Auditive -> ICT Knowledge	H1	0.167	0.000	Confirmed
Cognitive -> ICT Knowledge	H1	0.265	0.000	Confirmed
Visual -> ICT Knowledge	H1	0.197	0.000	Confirmed
Motor -> ICT Knowledge	H1	0.226	0.000	Confirmed
ICT Knowledge -> Motivation	H2	0.167	0.011	Confirmed
ICT Knowledge -> Use of ICT	H3	0.163	0.047	Confirmed
Use of ICT -> Motivation	H4	0.574	0.000	Confirmed

**Table 5:** Multi-group analysis.

Hypothesis	Path Coefficient		Difference	Path Coefficient	Path Coefficient
	Stage of Teaching		Coefficient	*p*-Value	*p*-Value
	Children’s and Primary Education	Higher Education		Children’s and Primary Education	Higher Education
WEB Accesability -> ICT Knowledge	0.238	0.237	−0.001	0.000	0.000
Auditive -> ICT Knowledge	0.159	0.174	−0.015	0.000	0.000
Cognitive -> ICT Knowledge	0.258	0.272	−0.014	0.000	0.000
Visual -> ICT Knowledge	0.186	0.206	−0.020	0.000	0.000
Motor -> ICT Knowledge	0.225	0.228	−0.003	0.000	0.000
ICT Knowledge -> Motivation	0.122	0.215	−0.093	0.193	0.047
ICT Knowledge -> Use of ICT	0.166	0.161	0.005	0.206	0.046
Use of ICT -> Motivation	0.565	0.576	−0.011	0.000	0.000

## Data Availability

Not applicable.

## Appendix A

**Table A1:** Items of the scales used.

**Knowledge of ICT in visual impairment**	
VISUAL1	I recognise different computer programmes specifically produced for the visually impaired.
VISUAL2	I can identify the subjects for which Perkins machines can be useful.
VISUAL3	I know how to create a written document in a word processor and eliminate the aspects that may make it difficult for visually impaired people to use it.
VISUAL4	In general, I am aware of the possibilities offered by ICT for visually impaired people.
VISUAL5	I am able to make ICT-supported curricular adaptations for visually impaired people.
**Knowledge of ICT for hearing impairment**	
AUDITIVE1	I am able to make ICT-supported curricular adaptations for hearing impaired learners.
AUDITIVE2	I know how sign language works
AUDITIVE3	In general, I am familiar with the possibilities offered by ICT for hearing impaired learners.
AUDITIVE4	I am able to apply ICT-supported teaching strategies to facilitate the integration of hearing impaired learners.
AUDITIVE5	I am able to point out different websites where a teacher can find educational resources for hearing impaired people.
**Knowledge about ICT in cognitive disability**	
COGNITIVE1	I am able to apply ICT-supported teaching strategies to facilitate the inclusion of learners with cognitive disabilities.
COGNITIVE2	In general, I am aware of the possibilities that ICT offers to subjects with cognitive disabilities.
COGNITIVE3	I can cite some educational programmes used for cognitive rehabilitation.
COGNITIVE4	I am able to make ICT-supported curricular adaptations for subjects with cognitive disabilities
COGNITIVE5	I am able to describe the main limitations that multimedia materials may contain for use with people with cognitive disabilities.
**Knowledge about ICT in motor disability**	
MOTOR1	I am familiar with different types of keyboards for people with different types of mobility limitations.
MOTOR2	I am generally aware of the possibilities offered by ICT for people with motor disabilities.
MOTOR3	I am familiar with specific software for people with motor disabilities.
MOTOR4	I am able to apply ICT-supported teaching strategies to facilitate the inclusion of learners with motor impairments.
MOTOR5	I am able to make ICT-supported curricular adaptations for people with motor impairments.
**Knowledge about ICT in web accessibility**	
WEB ACCESS1	I know what accessibility testing is for web sites
WEB ACCESS2	I am able to create web pages with high accessibility parameters.
WEB ACCESS3	I can point out different national and international institutions that are involved in the study and research of website accessibility.
WEB ACCESS4	I am able to explain the principles that the Design for All Centre recommends to follow in order to achieve websites that serve to achieve “design for all”.
WEB ACCESS5	I am able to cite different accessibility tests.
